# Mental health first aid training for nursing students: a protocol for a pragmatic randomised controlled trial in a large university

**DOI:** 10.1186/s12888-015-0403-3

**Published:** 2015-02-19

**Authors:** Gemma Crawford, Sharyn K Burns, Hui Jun Chih, Kristen Hunt, PJ Matt Tilley, Jonathan Hallett, Kim Coleman, Sonya Smith

**Affiliations:** 1 Collaboration for Evidence, Research and Impact in Public Health, School of Public Health, Faculty of Health Sciences, Curtin University, Perth, Australia; 2 School of Public Health, Faculty of Health Sciences, Curtin University, Perth, Australia; 3 School of Nursing and Midwifery, Faculty of Health Sciences, Curtin University, Perth, Australia

**Keywords:** University, Nursing students, Australia, Mental health literacy, Prevention and early intervention, Mental health first aid

## Abstract

**Background:**

The impact of mental health problems and disorders in Australia is significant. Mental health problems often start early and disproportionately affect young people. Poor adolescent mental health can predict educational achievement at school and educational and occupational attainment in adulthood. Many young people attend higher education and have been found to experience a range of mental health issues. The university setting therefore presents a unique opportunity to trial interventions to reduce the burden of mental health problems. Mental Health First Aid (MHFA) aims to train participants to recognise symptoms of mental health problems and assist an individual who may be experiencing a mental health crisis. Training nursing students in MHFA may increase mental health literacy and decrease stigma in the student population. This paper presents a protocol for a trial to examine the efficacy of the MHFA training for students studying nursing at a large university in Perth, Western Australia.

**Methods/Design:**

This randomised controlled trial will follow the CONSORT guidelines. Participants will be randomly allocated to the intervention group (receiving a MHFA training course comprising two face to face 6.5 hour sessions run over two days during the intervention period) or a waitlisted control group (not receiving MHFA training during the study). The source population will be undergraduate nursing students at a large university located in Perth, Western Australia. Efficacy of the MHFA training will be assessed by following the intention-to-treat principle and repeated measures analysis.

**Discussion:**

Given the known burden of mental health disorders among student populations, it is important universities consider effective strategies to address mental health issues. Providing MHFA training to students offers the advantage of increasing mental health literacy, among the student population. Further, students trained in MHFA are likely to utilise these skills in the broader community, when they graduate to the workforce. It is anticipated that this trial will demonstrate the scalability of MHFA in the university environment for pre-service nurses and that implementation of MHFA courses, with comprehensive evaluation, could yield positive improvements in the mental health literacy amongst this target group as well as other tertiary student groups.

**Trial registration:**

Australian New Zealand Clinical Trials Registry ACTRN12614000861651.

## Background

### Mental health and young people in Australia

The impact of mental health problems and disorders in Australia is significant. In the most recent national Australian mental health survey, almost half of respondents had experienced a mental health disorder at some point in their lives, and around one in five respondents aged 16–85 had experienced a mental health disorder in the preceding 12 months [1]. The most common mental health disorders are depressive, anxiety and substance use disorders, which are often comorbid [1].

Mental health disorders often start early with the first presentation for most mental health problems occurring between the ages of 18 and 29 [2]. Mental health problems also disproportionately affect young people [2] with 16–24 years olds experiencing the highest prevalence of mental health disorders than any other age group [3]. National data suggests that around 1 in 4 people aged 16–34 years had experienced a mental health disorder in the last 12 months [1] and that mental health disorders for those aged 15–44 years contributed to over a third (36%) of Australia’s Disability-Adjusted Life Years [4]. Common mental health disorders in young Australians aged 16–24 years are: anxiety disorders (15.4%), depressive disorders (6.3%) and substance use disorders (12.7%) [3].

### Mental health and university students

Poor adolescent mental health has been found to predict educational achievement at school and educational and occupational attainment in adulthood [5-9].This is of concern for the tertiary environment as over a quarter of young Australian adults aged 18 to 35 attend higher education [10] and increased levels of psychological distress may impair students’ academic capabilities in the university setting [11]. For example, a survey of US and Canadian college students (n = 71,860), found seven of the top ten health-related barriers to successful academic performance were related to mental health. In particular, stress was identified as the biggest barrier, affecting around a third of the students (33.9%); a diagnosis of either depression, anxiety disorder, or seasonal affective disorder was the 6th barrier (affecting 16.1% of students); and alcohol misuse was the tenth barrier (affecting 7.8% of students) [12]. Some studies indicate that mental health problems may be higher among university students compared to their non-tertiary student peers [13-18]. For example, a survey of two Australian universities found that most students (83.9%) reported higher levels of psychological distress when compared to the general population (29%) [11]. Further, students may not seek help for their mental health problems [14,19]. Reasons for this may include suicidal thoughts, perceived need for self-reliance, perceived stigma of talking to professionals, believing that nothing will assist, unfamiliarity or awareness of services or perception that stress is a normal part of study [20-22]. Financial strain and lower socio-economic status, or being a young or a female student may lead to higher levels of psychological distress, increasing the risk for poorer mental health [11,13,15,19,23-27]. This is significant as the Australian government aims for more, young, and socioeconomically disadvantaged people to complete a university qualification [28].

### Mental health literacy

Jorm et al. [29], suggested mental health literacy incorporates several components: “(a) the ability to recognise specific disorders or different types of psychological distress; (b) knowledge and beliefs about risk factors and causes; (c) knowledge and beliefs about self-help interventions; (d) knowledge and beliefs about professional help available; (e) attitudes which facilitate recognition and appropriate help-seeking; and (f) knowledge of how to seek mental health information” [29]. The most recent Australian survey of mental health literacy, undertaken in 2011, was compared to surveys taken in 1995 and 2003–4 [30]. It found mental health literacy among the general population had improved in every aspect excluding ‘stigmatising attitudes’ [30]. However, aspects such as ‘recognition of mental disorders’, ‘beliefs about prevention’ and ‘stigmatising attitudes’ were not significantly improved in young people [30]. Research has shown that improving the mental health literacy of a community not only decreases the stigma of mental health but also improves the mental health of community members [31]. Additionally, simple recognition of the name and a few characteristics of a mental health condition can decrease stigma towards those living with mental health problems and disorders [31]. Recognising a mental health disorder and lowering stigma also leads to more help seeking from relevant sources [32].

### Mental health literacy in the university setting

Whilst a number of interventions have been trialed to address mental health issues in universities [33-40], a greater focus on increasing mental health literacy may be valuable where mental health problems and stigmatising attitudes are common [41,42], and health literacy and help-seeking behaviours may be poor [41,43]. For example, a recent study implemented in an Australian university found that talking to a close friend or family member (as opposed to a health professional) was one of the most common forms of help seeking [42]. Another study found pharmacy students who participated in MHFA courses were better able to correctly identify mental illnesses and their treatments, held less stigmatising views toward mental illness, and reported greater confidence in delivering pharmacy services to those with a mental health problem [44].

### Training pre-service nurses

In Western Australia, there is a mental health workforce shortage across a range of disciplines [45]. Nurses constitute over half of Australian mental health healthcare providers and therefore play an important role in delivering quality early intervention and treatment for mental health disorders and problems [45].The attitudes of nurses towards mental health problems and those experiencing them are important as they have the potential to be stigmatising and discriminatory or very supportive [46]. Some sources have suggested that Australian nurses have low or even inadequate levels of mental health nursing skills when they enter the workforce [47,48]. Targeting students studying nursing may increase health literacy and decrease stigma in the student population at an early stage. It may also influence their attitudes towards mental health after graduation, encouraging best practice, client-centered care. A longitudinal study by McCann et al. found when nursing students begin their studies, they have a mental health literacy level analogous to that of the general public [47]. Further research by Ward suggested that structured training programs examining mental health issues increased nursing students’ confidence and preparedness to respond to mental health issues, and students indicated a desire for greater levels of education and training support [49].

### Mental health first aid

Mental health promotion, prevention and early intervention strategies have been shown to demonstrate positive outcomes [50] and have been a focus of Australian government policy for the past decade [51]. Given the high prevalence and burden of mental health disorders among student populations, trialing a range of interventions (universal, selective, indicated prevention and early intervention) [52] within the university setting to reduce negative mental health outcomes is important [39]. Mental Health First Aid courses were developed by Mental Health First Aid Australia (MHFA) in 2000 [53,54]. These courses primarily aim to train participants to recognise symptoms of mental health problems and assist someone who may be experiencing a mental health crisis. The course includes scenarios focusing on common mental health issues such as panic attacks, suicidal ideation, in addition to focusing on risk factors, signs and symptoms for mental health problems and disorders such as depression and anxiety [53,54]. In 2011, just ten years after commencement, 170,000 Australian adults had received MHFA training and over 850 instructors had been trained [55]. The MHFA program has been selected as one of 10 global initiatives to demonstrate “radical efficiency” due to its ability to deliver greater public outcomes than previous interventions at a reduced cost [56]. These courses have reportedly demonstrated increased mental health literacy and mental health of course participants and decreased stigma around mental health problems and disorders [57]. It is recognised that one of the challenges highlighted in the literature is that there are few studies that demonstrate the longer term impact of Mental Health First Aid on helping behaviours or participants’ subsequent experiences in providing first aid. However, those that have been conducted point to a positive outcome from Mental Health First Aid delivery in real-life practice though it has been suggested that this may require further exploration [58-61].

The Australian Government Department of Health has provided funding to Mental Health First Aid Australia [62] to specifically train frontline staff including financial counsellors and healthcare professionals (including pre-service nursing students) to better identify and respond to the needs of people at risk of mental health problems and disorders. The aim of the course developed for nursing students is to teach them how to assist a peer who is developing a mental health problem or in a mental health crisis. The course is available both as a face to face version and as an e-course online [63].

This paper presents a protocol for a pragmatic, low-cost trial to examine the efficacy of the MHFA training for students studying nursing at a large university in Perth, Western Australia. The primary outcome is changes in mental health literacy, which will be measured in terms of confidence in dealing with mental health problems, recognition of and knowledge about mental health problems, first aid intentions, stigmatising attitudes and social distance.

## Methods/Design

**Figure 1:**
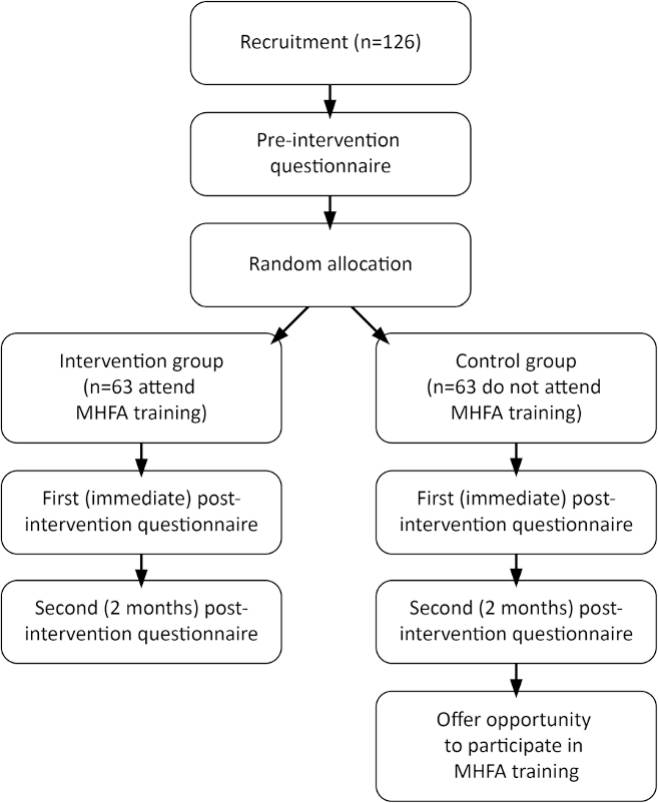
**Trial schema.**

This protocol proposes a randomised controlled trial to determine the efficacy of the MHFA training for university nursing students (see Figure 1). Participants will be randomly allocated to the intervention group (receiving MHFA training during the study) or the control group (not receiving MHFA training during the study and waitlisted to receive an online version of the course on completion of the intervention). Participants receiving the intervention will attend a MHFA course comprising two-6.5 hour face-to-face sessions run over two days. The study follows the CONSORT Guidelines [64] for the design and implementation of randomised controlled trials.

### Participants and sample size

The source population will be undergraduate nursing students at a large university located in Perth, Western Australia. Criteria for inclusion: 1) first year undergraduate nursing student; 2) enrolled in the internal study mode; 3) studying at the Curtin University Bentley campus; and 4) aged 18 years or older. The size of the sample has been determined by the practicalities of working in a large university which requires delivering the intervention during semester at a time convenient to university scheduling requirements and clinical rotations for the students. Assuming a high correlation of 0.8 for the baseline-post measurements [65,66], it will be necessary to have 50 students in each group to detect 5% level of significance with 90% power in order to detect medium effect sizes in the outcome variables [57]. A total of 63 students will be recruited for each group to account for 20% attrition from baseline to post intervention.

### Ethical approval

In order to eliminate any harm associated with not receiving the MHFA training, the control group will be offered an online version of the intervention at the end of this study after all participants have completed the two-month post intervention questionnaire. The online course covers the same content as the face to face course and takes around six to eight hours to complete. This study has been granted ethical approval by the Curtin University Human Research Ethics Committee, approval number: SPH-74-2013.

#### Stage 1: recruitment and group allocation

First year undergraduate nursing students will be contacted through email, fliers, and in-class announcements. Potential participants will be provided an information sheet and asked to provide consent if they agree to participate via the online survey. Students who agree to participate will complete a pre-intervention questionnaire and be randomly assigned to the intervention or control group through automated randomisation. Each participant will be provided with a de-identifiable code to ensure anonymity.

#### Stage 2: baseline questionnaire

Participants from the intervention and control groups will be asked to complete an online baseline questionnaire (occurring at the same time), which will be one week prior to the intervention. Participants will be emailed a link to the online survey. A reminder email will be sent once after the initial contact.

#### Stage 3: the intervention

The intervention group will receive Mental Health First Aid training. The MHFA training will consist of two 6.5 hour sessions, delivered face to face, which include:

- Signs, symptoms and risk factors of common mental health problems including depression, anxiety and substance use disorders, eating disorders and psychosis;
- Strategies to assist an individual who may be experiencing a mental health crisis such as a panic attack or suicidal thoughts or suicidal behavior; and
- What works to help those developing or experiencing a mental health problem or crisis [41].

Participants will be provided with standardised Mental Health First Aid materials, including the Mental Health First Aid Manual and additional tailored materials designed for nurses. Four courses of up to 20 students per course will be delivered over a period of two weeks by a team of three qualified MHFA facilitators who will include a mental health nurse and a community health promotion practitioner and a university academic. The third facilitator will provide consistency across all courses. The standardised MHFA training for nursing students will be piloted by the teaching team prior to the commencement of the intervention to ensure that the approach and delivery of the materials is consistent. Further information about the program has been described by the MHFA research team [54].

#### Stage 4: post-intervention questionnaire one

Immediately after course completion, the intervention group will be asked to complete the post intervention questionnaire in the classroom via i-pad. The control group will be emailed the same questionnaire at the same time. An email reminder will be sent twice a week for two weeks to those who do not respond in the first instance.

#### Stage 5: post-intervention questionnaire two

Two months after the MHFA course is delivered, the second post-intervention questionnaire will be emailed to all participants. An email reminder will be sent twice a week for two weeks to those who do not respond in the first instance.

#### Stage 6: MHFA for the control group

The waitlisted control group will be provided with access to the online version of the Mental Health First Aid training and will receive a copy of the MHFA training manual after the second post-intervention questionnaire is administered. Additionally, participants from the intervention group who complete the baseline questionnaire but do not complete the MHFA training will be provided an opportunity to undertake the online course at the completion of the intervention along with the control group.

#### Stage 7: process evaluation

Participant satisfaction with training content, materials, facilitators, venue and timing will be determined using a standard MHFA training evaluation form. Program reach and engagement will also be assessed by the initial participation rate and response rate at the end of the study.

#### Stage 8: follow up

A follow up will be conducted at 12 months. Participants from the intervention group will be invited to take part in a short follow up questionnaire to determine to what extent participants have maintained knowledge, skills and confidence and explore any opportunities they have had to use their skills and the impact that the training has had on this experience.

### Instruments

A self-report questionnaire will measure confidence to respond to mental health problems, recognition of and knowledge about mental health problems, first aid intentions towards someone experiencing a mental health problem, stigmatising attitudes [67] and social distance [68-70]. The questionnaire is based on those used in national surveys of adults [43] and young people [71] and further developed for use with the MHFA course [54,65,69], and described in detail in previous studies [44,57,72]. Questions related to confidence, recognition, intentions, stigmatising attitudes and social distance will be introduced with a vignette describing a young person with symptoms of a mental health problem. The use of the vignette to introduce mental health issues has been used successfully by other studies, including those of MHFA [44,57,68,72]. Participants will be asked about their level of confidence to help and what they would do to assist the person in the vignette [65]. Knowledge will be measured using 16 questions developed by MHFA and modified for use with young people [66]. Stigmatising attitudes will be measured using personal and perceived stigma scales [67], and social distance will be measured using a five item social distance scale [68]. The scales have been used widely and previously tested for reliability and validity; the stigma scales have demonstrated test-retest reliability and acceptable internal consistency while the social distance scale has been shown to be a reliable measure of stigma towards people with mental health problems and to be valid as a proxy measure for rejection of and discrimination toward people with mental health problems and disorders [67,69,70,73]. The questionnaire will be administered to the intervention and control group participants at three time intervals: baseline, immediately post, and two months after the intervention. Basic socio-demographic characteristics of participants will be collected at baseline only.

In addition, the questionnaire administered immediately post training will collect process evaluation data to measure participant satisfaction with the relevance and calibre of the MHFA training. A follow up questionnaire will be administered at 12 months post study with participants from the intervention group to explore participant experiences with using knowledge and skills from the training, interactions with individuals they thought may have a mental health problem; and for those who had not had an opportunity to use their training directly, gauge expectations, intentions and confidence to provide help.

### Statistical analyses

The intention-to-treat principle will be followed, where all participants who completed the baseline questionnaire will be included in the analysis regardless whether they complete the whole intervention. Acknowledging there may be attrition at the follow-up, the missing values for the non-respondents will be recorded using their baseline scores. If the missing data are at random, the imputation method will be applied [59]. This will assure no false improvement in efficacy. Data will be analysed using SPSS Version 22.

The demographics of the participants in the intervention and control groups will be compared using the independent samples t-test or Pearson’s Chi-square test to measure differences including age, gender and country of birth. The primary outcome measures (described above) will be calculated following validated MHFA scoring methods reported in the literature [44,54,57,65-68]. Repeated measures analysis of variance will be employed to assess continuous measures between the intervention and control group over three time points (baseline, post intervention and two months post intervention). Logistic regression will be used to assess changes in dichotomous variables. The significance level will be set at 5%.

## Discussion

Trialing strategies to reduce the burden of mental health problems and disorders in the setting where students socialise, live and study is important and will likely influence their attitudes post university towards mental health and wellbeing and those living with mental health problems and disorders. Due to the voluntary nature of participation in this intervention, selection bias may occur, however the randomisation of the treatment allocation used in this study attempts to reduce this effect. Findings including the efficacy and implications from this study will therefore provide information supporting the early implementation of MHFA training for nursing students to increase their mental health literacy and thus potentially improve future mental health care delivery. MHFA training has limited research regarding its implementation in young adults. If this intervention demonstrates the scalability of MHFA in the university environment for pre-service nurses, it could provide support for teaching mental health first aid across a wider variety of university courses. It is anticipated that implementation of MHFA courses, with comprehensive evaluation, could yield positive improvements in the mental health literacy amongst this target group.
